# Centers for Medicare & Medicaid Services Approval of Cardiac Ablations in Ambulatory Surgery Centers: Challenges and Emerging Models Shaping the Future of Cardiac Electrophysiology

**DOI:** 10.19102/icrm.2026.17015

**Published:** 2026-01-15

**Authors:** Arash Aryana, Vijendra Swarup

**Affiliations:** 1 Mercy General Hospital and Dignity Health Heart and Vascular Institute, Sacramento, CA, USA; 2 The Arizona Heart Rhythm Center, Phoenix, AZ, USA

**Keywords:** Ambulatory surgery center, ASC, atrial fibrillation, catheter ablation, Centers for Medicare & Medicaid Services.

Over the past decade, ambulatory surgery centers (ASCs) have expanded across a wide range of specialties by offering lower costs, greater efficiency, and a superior patient experience as compared to hospital outpatient departments (HODs).^1–3^ As a result, various cardiovascular procedures, including cardiac implantable electronic devices, coronary angiography, elective percutaneous coronary intervention, and many vascular interventions, have steadily migrated into ASC settings. Although cardiac ablation had historically lagged behind this trend, the recent decision by the US Centers for Medicare & Medicaid Services (CMS) to add several key ablation codes to the ASC Covered Procedures List represents a pivotal shift in the site-of-care landscape for cardiac electrophysiology (EP).^4^ This commentary summarizes the clinical evidence, operational requirements, economic considerations, and strategic implications of performing cardiac ablation in ASCs, offering a clear and practical framework for cardiac electrophysiologists and health systems navigating this transition.

## Background

The current ASC model originated in 1970, when two Arizona physicians, Wallace Reed and John Ford, established the first freestanding center offering same-day procedures—an innovation that invoked a trend in the national expansion of outpatient procedures and surgeries.^5^ Since the 1980s, ASC growth has been steady, with dozens of new centers opening annually. Today, more than 6300 Medicare-certified ASCs perform over 23 million procedures each year.^5^ The CMS has approved roughly 3500 procedures for ASC reimbursement, and this list continues to grow.

ASCs typically operate around focused service lines and are designed to provide hospital-level care, albeit with greater efficiency and lower overhead. These advantages translate into substantial system-wide savings. A 2023 BlueCross BlueShield Association report found that HOD-based procedures remain significantly more costly than those performed in ASCs. Increasingly, commercial payers also incentivize ASC utilization through more favorable reimbursement arrangements.^6^

## Catheter ablation: The scope of the problem

Catheter ablation is increasingly used as early or first-line therapy for many cardiac arrhythmias, including atrial fibrillation (AF). Procedural volumes continue to rise due to improved safety and efficacy; expanded indications; and rapid technological advances in mapping, imaging, and ablation. Meanwhile, many US hospitals face capacity constraints, resulting in prolonged wait times that pose clinical consequences. Delays in catheter ablation are associated with increased risks of mortality, heart failure, stroke (12.2%), hospitalization (11.8%), emergency department use (30.9%), and higher overall health care spending.^7,8^ With catheter ablation volumes projected to grow 14% annually between 2025 and 2030—doubling over 8 years—capacity challenges will intensify unless alternative sites of service, including ASCs, are fully leveraged.^9^

## Same-day discharge after catheter ablation

Advances in cardiac ablation technology and standardized care pathways have made same-day discharge routine for most patients. Many academic and community hospitals now achieve same-day discharge rates exceeding 90%.^10^ A substantial body of evidence, including CMS claims analyses and multicenter observational studies, supports these practices.^11,12^ Across more than 200,000 evaluated patients, studies comparing same-day discharge with overnight hospital monitoring demonstrate no differences in complication rates, readmissions, or emergency department visits. The consistency of these findings across diverse populations reinforces the safety and scalability of outpatient ablation workflows.

## Catheter ablations in ambulatory surgery centers

The migration of cardiac ablation to ASCs represents the next logical step in cardiovascular care delivery.^13^ ASC-based ablation can expand access, reduce exposure to hospital-acquired infections, and lower system-wide costs.^14^ In November 2025, the CMS finalized the 2026 Hospital Outpatient Prospective Payment System and ASC Payment System rule, adding the codes for catheter ablation of atrioventricular node, supraventricular tachycardia, AF, and ventricular arrhythmias as well as cardioversion to the ASC Covered Procedures List.^4^

This policy change followed multiple published studies demonstrating the safety and feasibility of ASC-based ablation, including multicenter series totaling more than 4000 cases.^15–20^ It should be emphasized that contemporary data show major adverse event rates of <1% for cardiac ablation with current National Cardiovascular Data Registry (NCDR) benchmarks at 0.6%—nearly 10-fold lower than two decades ago.^21–23^ For context, the NCDR CathPCI Registry reports a 1.83% major adverse event rate for percutaneous coronary intervention—a procedure currently approved and frequently performed in ASCs across the United States.^24^ Total hip and knee arthroplasty complication rates also range between 1.8% and 9%.^25,26^ CMS’s approval of the cardiac ablation codes, therefore, represents not only a regulatory milestone but also an evidence-based endorsement. Economic analyses further suggest substantial savings for payers and reduced out-of-pocket burden for patients, improved affordability, and equity in rhythm management.^14^

## Emergence of cardiac electrophysiology–capable ambulatory surgery centers

The trends observed in orthopedic surgery and gastrointestinal ASCs offer a preview of the likely evolution in cardiac EP, wherein emerging technologies enable outpatient migration, standardized protocols ensure safety, CMS expands coverage, and commercial payers accelerate adoption. Cardiac EP is currently following the same trajectory; yet, several barriers remain. State regulations vary widely, and certificate-of-need rules may limit expansion. While some states permit cardiovascular procedures, others restrict interventions requiring hemodynamic monitoring. Additional challenges include transfer agreement requirements, the capital intensity of EP laboratories, specialized staffing, and reimbursement gaps for select technologies.

The development of cardiac EP–capable ASCs, therefore, requires coordinated planning across multiple domains, as follows:

1. ***Infrastructure.*** EP laboratories equipped with fluoroscopy, intracardiac and transesophageal echocardiography, mapping and ablation platforms, radiation shielding, crash carts, and resuscitation equipment.
2. ***Operations.*** Electronic medical record systems, ASC management, billing and coding, payer contracting, supply chain management (including reprocessed products and devices), and emergency pathways.
3. ***Staffing.*** Nurses, technicians, and anesthesia teams with EP-specific expertise and familiarity with same-day discharge workflows.
4. ***Data.*** Systems to track success rates, 30-day readmissions, major adverse events, transfers, and patient-reported outcomes.

## Ambulatory surgery center ownership models

Ownership of ASCs generally involves three key stakeholder groups: (1) physicians, (2) ASC management companies, and (3) hospital systems, resulting in five common equity models^27^:

1. ***Sole physician ownership.*** Physicians hold 100% equity, providing full authority over governance and operations, retaining complete control over all decisions and distributions. Management services may be contracted without relinquishing equity. This model still remains the most common nationally.
2. ***Physician–management company joint venture.*** Either party may be the majority owner. Physicians retain clinical control while benefiting from management expertise, contracting, and revenue cycle support.
3. ***Physician–hospital joint venture.*** Hospitals are increasingly pursuing this type of model to retain the outpatient market share. Physicians may benefit from improved payer contracting and financial stability.
4. ***Three-way joint venture.*** A shared ownership model among physicians, management firms, and hospitals, in which all parties contribute expertise. Majority ownership may be shared to maintain balance and alignment.
5. ***Sole hospital ownership.*** Hospitals operate ASCs independently, often with physician co-management agreements. This type of model seems to be growing nationally.

## Successful blueprints for cardiac electrophysiology ambulatory surgery centers

Despite growing participation from hospitals and corporate entities, physician ownership continues to remain central to ASCs, such that 90% include some degree of physician ownership and 65% are fully physician-owned.^28^ Compliance with Stark Law safe harbors is essential. Physician-owners must perform at least one-third of their procedures in the ASC, purchase equity at fair market value, and receive distributions strictly according to ownership percentage.^28^ Operationally, physician-owned ASCs must manage contracting, staffing, financial oversight, and regulatory compliance, which may necessitate external management support. As this can dilute ownership, many physicians often prefer the autonomy, efficiency, and patient-centric culture of physician-led ASCs.^29^

## What is currently missing?

Although widespread interest in EP-dedicated ASCs has continued to expand, cardiac electrophysiologists have lacked centralized, affordable, specialty-aligned resources to guide the development and operations of such entities. Instead, most rely on fragmented consultants, management companies, or vendors, each addressing only narrow components of the complete process. This fragmentation not only increases cost but also slows development, reduces efficiency, and can shift strategic control away from physicians. In many scenarios, external partners assume disproportionate influence or ownership, ultimately diminishing physician control and involvement.

Hence, what the field avidly needs is a physician-governed, nationally coordinated, specialty-aligned framework that provides organized, end-to-end support for developing EP-capable ASCs—without taking on corporate ownership! Such an organization would preserve physician autonomy while supplying the operational, regulatory, and technical capabilities required for safe and scalable growth. This includes EP-specific facility design tailored to EP workflows, equipment and capital planning, accreditation and compliance pathways, safety protocols, revenue cycle optimization and contracting support, data infrastructure, and cost-efficient supply chains. Incorporating modern artificial intelligence–enabled documentation, scheduling, and cost analytics would further reduce administrative burden and strengthen financial sustainability. Just as importantly, this structure would provide a counterbalance to large management firms or health systems whose involvement—while sometimes helpful—can come at the expense of physician ownership or long-term strategic control. An affordable, coordinated, and physician-led model would help ensure that cardiac electrophysiologists retain clinical control and economic authority over their ASCs, while expanding access to high-quality outpatient cardiac EP and ablation services nationally.

## The proposed model

An emerging example of such a coordinated, physician-governed framework is the ACCESS ecosystem. ACCESS includes two complementary structures: (1) the ACCESS Foundation, which is a nonprofit entity dedicated to EP-specific ASC education, safety standards, regulatory training, workforce development, and best-practice guidelines, and (2) ACCESS, Inc., which is an operational arm providing non-equity support, such as compliance pathways, accreditation readiness, digital workflow tools, revenue cycle optimization, and supply chain coordination. Together, these complementary entities aim to preserve physician control and ownership, promote transparent and standardized workflows, and create a unified national framework for the safe and sustainable adoption of cardiac EP–capable ASCs.

In short, the growth of EP ASCs requires more than policy change and clinical feasibility. It requires a unified support system built to protect physician leadership to ensure sustainable, scalable, and equitable development across the cardiac EP specialty. Disruptive models coordinated nationally and led by physicians are necessary and will likely play an important role in shaping the future and evolution of cardiac EP.

## Conclusion

ASCs can offer a safe, efficient, and clinically validated setting for many cardiac EP procedures, including cardiac ablation. Technological advancements, standardized workflows, and robust evidence supporting same-day discharge demonstrate that complex EP care can be delivered outside hospitals without compromising safety or quality. Following recent CMS policy changes, economic and regulatory incentives now strongly support the expansion of cardiac EP ASCs.

Yet, the remaining challenges are primarily infrastructural and organizational. Consistent regulatory frameworks, standardized operational models, and physician-centered support structures will determine the pace and safety of nationwide ASC adoption in EP. Without such frameworks, development may remain fragmented, costly, and at risk of eroding physician control. Physician-led models, such as the ACCESS Foundation and ACCESS, Inc., offer a promising pathway for safe and sustainable expansion. Addressing current gaps will enable EP ASCs to meet the rising procedural demands, reduce wait times, enhance patient experience, lower system costs, and preserve physician leadership in the next era of cardiovascular and arrhythmia care.
